# Erectile dysfunction as a marker of endocrine and glycemic disorders

**DOI:** 10.1007/s40618-022-01788-5

**Published:** 2022-04-02

**Authors:** R. Mazzilli, V. Zamponi, S. Olana, N. Mikovic, D. Cimadomo, G. Defeudis, A. Faggiano

**Affiliations:** 1 Department of Clinical and Molecular Medicine, University “Sapienza” of Rome, Sant’Andrea Hospital, Via di Grottarossa 1038, 00189 Rome, Italy; 2 GeneraLife IVF, Clinica Valle Giulia, via G. de Notaris 2b, Rome, Italy; 3 Unit of Endocrinology and Diabetes, Department of Medicine, University Campus Bio-Medico di Roma, Rome, Italy

**Keywords:** Erectile dysfunction, Diabetes mellitus (DM), Sexual function, Hypogonadism, Thyroid dysfunction, Prolactin

## Abstract

**Purpose:**

The aim of this study was to evaluate in a population of patients with erectile dysfunction (ED): (a) the prevalence of a previously unknown endocrine/glycemic disorders; (b) the correlation between ED severity and endocrine/glycemic disorders.

**Methods:**

1332 patients referred for ED from 2013 to 2020 were included. The ED diagnosis was made using the International-Erectile-Function-Index-5 questionnaire. ED severity was considered according to presence/absence of spontaneous erections, maintenance/achievement deficiency. All patients were subjected to search for sociodemographic and clinical characteristics: age, ethnicity, marital status, previous use of PDE5i, previous prostatectomy, diabetes mellitus (DM), prediabetes, endocrine dysfunctions.

**Results:**

The mean ± SD age was 54.3 ± 13.7 years. The 19.1% (255/1332) of patients were already in treatment for prediabetes/diabetes or endocrine dysfunctions. Among the remaining 1077, the prevalence of previously unknown endocrine and glycemic disorders was 30% (323/1077). Among them, 190/323 subjects (58.8%) were affected by hypogonadism, with high estradiol level observed in 8/190 (4.2%). The prevalence of new glycemic alterations was 17.3% (56/323) [specifically, 32/56 (57.1%) DM, and 24/56 (42.9%) prediabetes]. A thyroid dysfunction was observed in 40/323 subjects (12.3%) and hyperprolactinemia in 37/323 (11.5%). Patients with new diagnosis of DM showed more severe form of ED compared to the total group {difficulty in the achievement of erection: 46/56 [82.2%, vs 265/1332 (19.9%), *p* < 0.05]; absence of spontaneous erection 23/56 [41.1%, vs 321/1332 (24.1%), *p* < 0.05]}.

**Conclusion:**

ED is an early marker of endocrine/glycemic disorder, and a previously unknown dysfunction was found in more than a quarter of patients. A newly diagnosed DM is associated with ED severity, especially in elderly man and in presence of hypertension.

## Introduction

Erectile dysfunction (ED), defined as the inability to maintain or to get an erection firm enough to have sexual intercourse, is a multidimensional male sexual dysfunction [1]. The prevalence of ED is ranging from 6 to 64%, depending on different group considered, age and comorbidities, with an average prevalence of 30% [2, 3].

ED involves organic, relational as well as stress and mental health concerns [4]. In fact, sexual health is strictly related with general health as well as psychosexological factors, even if psychological symptoms are seldom investigated [4]. Among endocrine disorders, hypogonadism, hyperprolactinemia, thyroid dysfunction, as well as diabetes mellitus (DM) and impaired glucose tolerance are involved in ED development [5]. Although the International Committee for Sexual Medicine do not routinely suggest hormonal investigation in men with sexual dysfunction, apart from testosterone and prolactin, because of the current lack of convincing evidence [6], several studies highlighted that the management of endocrine disorders in patients with ED is useful, to establish an appropriate therapeutic strategy and ensure complete restoration of symptoms [5, 7]. The most frequent endocrine alteration affecting male sexual function is represented by hypogonadism. Testosterone is crucial in the regulation of the expression of nitric oxide synthase (NOS) and phosphodiesterase type 5 inhibitors (PDE5i) inside the penis [7]; testosterone modulates erectile function and regulates the timing of the erectile process and sexual desire. The prevalence of ED due to hypogonadism ranges from 2 to 21% [8]. Hyperprolactinemia could also lead to sexual dysfunction, due to the inhibition of gonadotropin-releasing hormones, which, in turn, decreases the secretion of testosterone [5]. On the other hand, type 2 diabetes mellitus (T2DM) is the second most common risk factor for ED, which occurs three times more frequently in diabetics than non-diabetics, (about in 50–75% of males with DM) [9–11], by acting on macrovascular (cardiovascular events), microvascular, as well as neural system. It is well established that ED is an early marker of endothelial damage and hyperglycemia and results to be the first sign of DM in about 12–15% of patients [12, 13]. To this regard, Corona et al. [14] showed a higher risk of severe ED and pathological penile Doppler ultrasound parameters in patients with DM, both established and newly diagnosed, as well as impaired fasting glucose. On the other hand, the wide and early use of PDE5i in the natural history of ED, rapidly improving the erectile function, has led to a significant number of missed diagnoses of underlying this symptom [15].

However, apart from DM, most of the studies take in account the prevalence of endocrine disorders already known at the time of ED diagnosis [2, 5, 10].

The aim of this study was to evaluate the prevalence of a previously unknown glycemic and endocrine disorder, in a population of patients with ED and to evaluate if a relationship exists between specific endocrine disorders and ED severity, evaluated as presence/absence of spontaneous erections as well as maintenance/achievement deficiency.

## Materials and methods

This retrospective observational study included a total of 1782 subjects, referred consecutively to the Andrology Unit (Sant’Andrea Hospital—“Sapienza” University of Rome) for a condition of ED from January 2013 to January 2020.

Inclusion criteria were: (1) male sex; (2) presence of ED; (3) age between 18 and 75 years. Exclusion criteria were: (1) previous prostatectomy or other surgery in the pelvic region; (2) psychiatric disease in treatment with psychotropic drugs; (3) other sexual or ejaculatory dysfunction.

Sociodemographic and clinical characteristics were recorded: age, ethnicity, marital status, previous use of PDE5i, previous prostatectomy, diabetes mellitus, prediabetes disorders, endocrine dysfunctions.

The andrological examination aimed at evaluating the testis (shape, size, and appearance), epididymis, penis, and body hair distribution, and the possible presence of gynecomastia. The diagnosis of ED was made using the International Index of Erectile Function-5 (IIEF-5) questionnaire. IIEF-5 is a validated and reliable short form questionnaire to identify erectile dysfunction (ED: total score ≤ 21) [16]. ED severity was considered according to presence of spontaneous erections (that means an increase in blood flow in the corpora cavernosa and a contraction of the ischiocavernosus and bulbospongiosus muscles [17]), or absence, as well as maintenance/achievement deficiency, evaluated by the questions n.2 and n.3 of the IIEF-5 [16].

Blood samples were obtained at 8:00am; the plasma levels of luteinizing hormone (LH), follicle stimulating hormone (FSH), thyroid stimulating hormone (TSH), testosterone, estradiol, prolactin, HbA1c were measured. Chemiluminescence microparticle immunoassay (CMIA) and immunoassay (CLIA) were used.

Diagnosis of glycemic and endocrine disorder was made through hormonal and biochemical evaluation, as follows: (1) hypogonadism: total testosterone < 2.64 mg/dl, or total testosterone between 3.5 and 2.64 mg/dl, with free testosterone < 6.5 ng/dl [18]; (2) hyperprolactinemia: prolactin levels > 25 ng/ml, measured after a rest of at least 20 min after insertion of a cannula; (3) overt thyroid hyperthyroidism or hypothyroidism: TSH < 0.3 ulU/ml or TSH > 10 ulU/ml or subclinical conditions: TSH between 0.3 and 0.5 or between 5 and 10 ulU/ml with normal serum free thyroxine (FT4) [19, 20]; (4) high estradiol level: estradiol > 40 pg/ml (5) prediabetes: HbA1c: 42–47 mmol/mol (6.0–6.4%); (6) DM: HbA1c: ≥ 48 mmol/mol (≥ 6.5%) [21].

The study adhered to the Hospital’s Ethics Committee guidelines and to the Ethical Principles for Medical Research Involving Human Subjects as adopted at the 18th WMA General Assembly, Helsinki, Finland, June 1964, and amended by the 55th WMA General Assembly, Tokyo, Japan, October 2004 and subsequent modifications when enforced (last, Fortaleza, Brazil, October 2013).

### Statistical analysis

Continuous data were described as absolute values, mean ± standard deviation (SD), and range. Categorical data were described as absolute number and percentage frequency. We used Fisher’s exact test for analyzing categorical data and paired t-test for continuous data; *p* < 0.05 was considered statistically significant. The software R version 2.14.2 (Free Software Foundation, Inc., USA) was used for statistics and logistic regression analyses.

Sample-size calculation indicated that recruitment of 178 participants would be required to achieve 95% power. Based on previous studies [2, 3], these calculations assumed a prevalence of ED of 30% in the general population compared with an estimated prevalence of 50% in a population affected with endocrine/glycemic disorders (*α* error, 0.01; *β* error, 0.2).

## Results

**Fig. 1:**
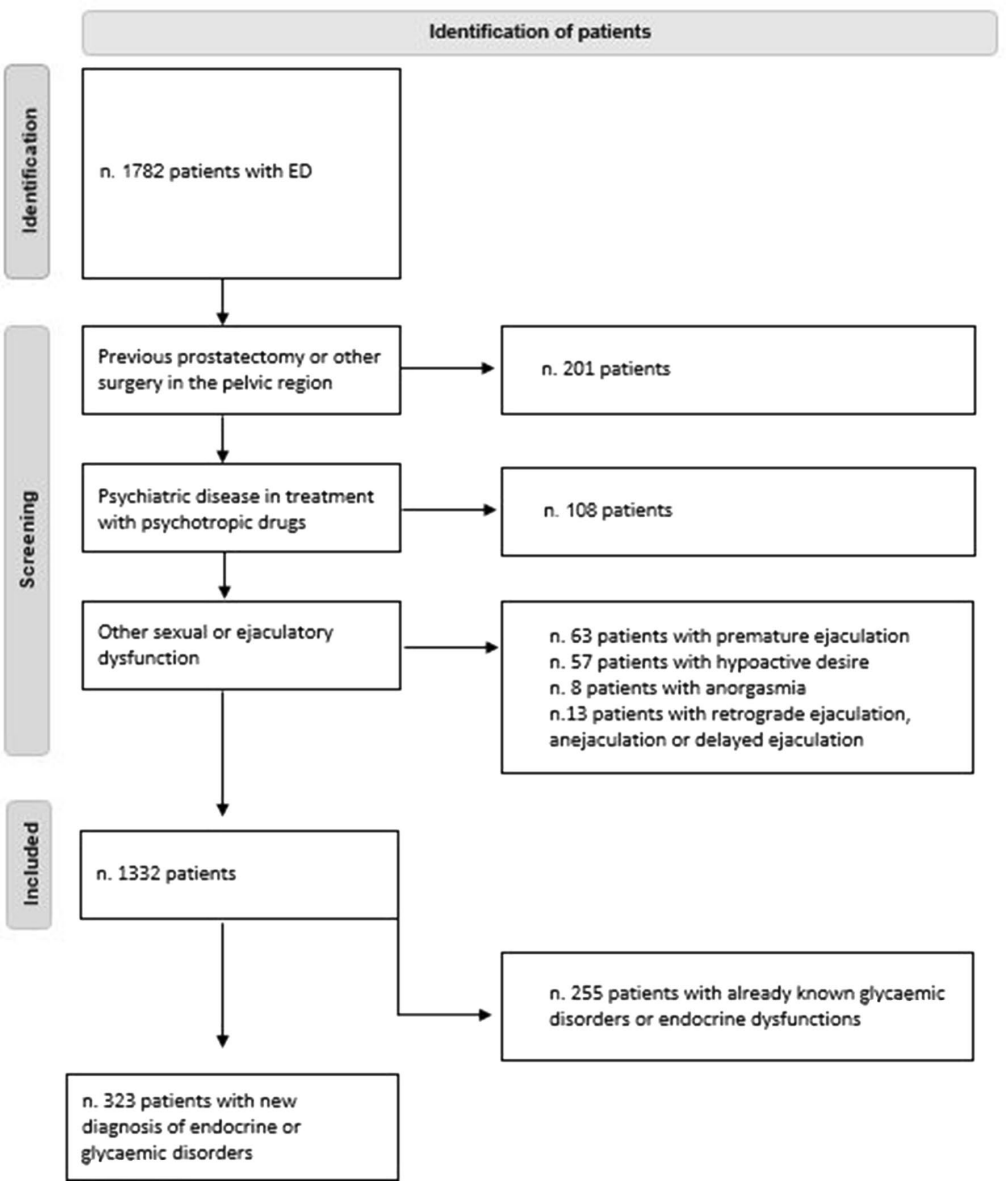
Flowchart of patient enrollment

A total of 1782 patients with ED were evaluated. Of them, 201 patients were excluded due to a previous prostatectomy or other surgery in the pelvic region; 108 patients because they are taking psychotropic drugs; 63 patients because suffered from premature ejaculation, 57 from hypoactive desire, 8 from anorgasmia and 13 retrograde ejaculation, anejaculation or delayed ejaculation. Thus, the final sample size included 1332 patients (Fig. 1).

The main characteristics of these patients are summarized in Table 1. Overall, the mean ± SD age was 54.3 ± 13.7 years. A rate of 88.3% of the patients had a stable relationship; the 80.1% of patients (1067/1332) referred difficulty in the maintenance of the erection, while the 19.9% (265/1332) in the achievement. The spontaneous erections were absent in 24.0% of patients, sporadic in 50.7% and present in the remaining 25.2%. A total of 386 patients had previously used at least one type of PDE5i, with efficacy in 51.6% of them.

**Table 1:** Clinical, hormonal and glycometabolic profile in the total group

Characteristics	Values
Age (years; mean ± SD)	54.3 ± 13.7
Status	
Single (*n*, %)	156 (11.7)
Relationship (*n*, %)	269 (20.2)
Married (*n*, %)	907 (68.1)
Spontaneous erection	
Absence (*n*, %)	321 (24.0)
Sporadic (*n*, %)	676 (50.8)
Normal (*n*, %)	335 (25.2)
Type of ED	
Maintenance (*n*, %)	1067 (80.1)
Achievement (*n*, %)	265 (19.9)
Testosterone (ng/ml; mean ± SD)	4.4 ± 1.6
Estradiol (mean ± SD)	32.0 ± 13.5 (2.7–61.0)
FSH (U/l; mean ± SD)	7.3 ± 7.9 (0.3–78)
LH (U/l; mean ± SD)	5.2 ± 4.3 (0.1–45.1)
PRL (ng/ml; mean ± SD)	10.0 ± 6.0 (0.5–51)
TSH (U/ml; mean ± SD)	1.8 ± 1.8 (0.001–24.7)
PSA (ng/ml; mean ± SD)	1.4 ± 1.6 (0.1–13)
HbA1c (%; mean ± SD)	6.2 ± 1.3 (4.0–12.0)
Estradiol (mean ± SD)	32.0 ± 13.5 (2.7–61.0)
Triglycerides (mg/dl; mean ± SD)	177.2 ± 134.0 (45.1–222.0)
Total cholesterol (mg/dl; mean ± SD)	187.2 ± 141.3 (98.2–251.3)

*ED* erectile dysfunction, *PRL* Prolactin, *TSH* thyroid stimulating, *PSA* prostate specific antigen, *FSH* follicle stimulating hormone, *LH* luteinizing hormone

A total of 255/1332 (19.1%) of patients had been already diagnosed with an endocrine or glycemic disorder, also receiving a specific therapy. Among these patients, 210 (82.4%) were diabetic, 12 (4.7%) were hypogonadic, 4 (1.6%) were affected by hyperprolactinemia and 29 (11.4%) suffered from hypo- or hyperthyroidism.

Among the remaining 1077 patients, the prevalence of subjects with unknown endocrine disorders was 30% (323/1077); none of these patients presents other specific signs or symptoms. The biochemical parameters are described in Table 2.

**Table 2:** Hormonal and HbA1c values in patients with new diagnosis of endocrine alteration or diabetes

	Age (years)	Testost. (ng/ml)	Estradiol (mg/dl)	FSH (U/l)	LH (U/l)	PRL (ng/ml)	TSH (U/ml)	HbA1c (%)
Diabetes	61.7 ± 10.3	3.8 ± 1.1	29.0 ± 2.8	8.0 ± .1	6.1 ± 6.5	8.5 ± 4.8	1.8 ± 0.9	7.6 ± 1.5
Hypogonadism	54.1 ± 12.8	1.9 ± 0.5	31.7 ± 16.1	9.1 ± 13.2	6.2 ± 8.2	10.5 ± 6.4	1.7 ± 0.8	5.9 ± 0.4
Hyperthyroidism	69.2 ± 5.7	4.2 ± 0.7	26.0 ± 5.7	9.5 ± 3.9	3.8 ± 2.8	10.7 ± 5.6	< 0.0 ± 0.02	5.5 ± 0.3
Hypothhyroidism	52.8 ± 12.5	3.9 ± 1.3	28.5 ± 3.5	5.3 ± 2.4	3.7 ± 1.5	9.0 ± 4.9	16.9 ± 5.6	5.5 ± 0.6
Hyperprolactinemia	44.5 ± 12.9	4.6 ± 1.9	30.5 ± 14.8	9.7 ± 11.5	7.5 ± 7.6	26.1 ± 7.3	2.1 ± 1.1	5.6 ± 0.7

*PRL*  Prolactin, *TSH* thyroid stimulating, *FSH* follicle stimulating hormone, *LH* luteinizing hormone

Overall, 190/323 subjects (58.8%) were diagnosed as affected by hypogonadism. Of them, 66 (34.7%) showed total testosterone levels < 2.64 ng/ml, while the remaining 124 (65.3%) total testosterone between 3.5 and 2.64 ng/ml, with free testosterone < 6.5 ng/dl. In 72.7% of the cases, the gonadotropins were within the normal range, while in the remaining cases (27.3%) were high. In 8/190 (4.2%) hypogonadal patients, high level of estradiol were also observed.

The prevalence of new glycemic alterations was 17.3% (56/323). Specifically, 32/56 (57.1%) were affected with DM, while 24/56 (42.9%) with prediabetes.

A thyroid dysfunction was assessed in 40/323 subjects (12.3%). In particular, in 4/40 patients (10.0%) there was overt hypothyroidism, in 10/40 (24.0%) subclinical hypothyroidism, in 4/40 patients (10.0%) overt hyperthyroidism and in 22/40 (55.0%) subclinical hyperthyroidism. Finally, 37/323 subjects (11.5%) were hyperprolactinemic.

A rate of 27.9% (90/323) of patients with newly diagnosis glycemic or endocrine dysfunction referred the use of PDE5i at least one time, with a positive response in 52.2% of the cases (47/90; *p* = 0.73 vs total group). Of them, 59.2% (58/98) received the prescription from their General Practitioner, while the remaining 40.8% of the patients bought online or received from friends and/or relatives.

Among the subgroups, patients with hyperprolactinemia were younger as compared to the total group (44.5 ± 12.9 vs 54.3 ± 13.7 years; *p* < 0.05). Furthermore, among patients with new diagnosed pathologies, BMI was significantly higher in patients with DM (26.2 ± 3.3 kg/m^2^) compared to thyroid dysfunction (24.5 ± 2.1 kg/m^2^; *p* = 0.002) and hyperprolactinemia (24.1 ± 1.8 kg/m^2^; *p* = 0.0001), and in patients with hypogonadism (25.6 ± 3.1 kg/m^2^) compared to thyroid dysfunction (*p* = 0.006). Similarly, the prevalence of hypertension was significantly higher in patients with new diagnosis of DM (31/56, 55.4%) compared to thyroid dysfunction (9/40, 22.5%; *p* = 0.002) and hyperprolactinemia (7/37, 18.9%; *p* = 0.0005), and in patients with hypogonadism (84/190, 44.2%) compared to thyroid dysfunction (*p* = 0.01) and hyperprolactinemia (*p* = 0.005).

By evaluating the relationship between severity of ED and previously unknown endocrine disorders, patients with newly diagnosed DM showed more severe form of ED as compared to the total group [difficulty in the achievement of erection: 46/56 (82.2%) vs 265/1332 (19.9%), *p* < 0.05; absence of spontaneous erection 23/56 (41.1%) vs 321/1332 (24.1%), *p* < 0.05]. A logistic regression analysis was performed to evaluate the effect of confounders on the severity of ED; the results showed that new diagnosis of diabetes (OR 3.3, 95% CI 1.3–8.2; *p* = 0.009), the presence hypertension (OR 3.3, 95% CI 1.3–8.2; *p* = 0.001) and age (OR 1.03, 95% CI 1.02–1.04; *p* = 0.001) affect the presence/absence of spontaneous erection, but not BMI (*p* = 0.8), while the difficulty in the achievement of erection was affected only by new diagnosis of diabetes (*p* < 0.001).

Finally, the mean ± SD of HbA1c was similar in the group with already known diabetes (7.3 ± 1.5%) compared to the group of new diagnosis 7.6 ± 1.5% (*p* = 0.3).

## Discussion

It is well known that ED can result from pre-existing hormone and metabolic impairments. At the opposite, ED can be an early marker of latent endocrine disorders. If this correlation has been established for DM, no data are available for other endocrine disorders, potentially associated with ED. In order to increase the convincing evidence for performing hormonal investigation in men with sexual dysfunction, we studied the prevalence of a previously unknown glycemic and endocrine disorder, in a population of patients with ED.

In this study, in 30% of subjects affected by ED, a new diagnosis of a previously unknown endocrine disorder was carried out. Hypogonadism was the most frequent hormonal alteration in the group of subjects with new diagnosis of endocrine/metabolic disorders. This is not surprising, since androgens play a central role in enhancing sexual desire and maintaining adequate sleep-related erections [5, 9]. Again, testosterone modulates nearly every component involved in erectile function, including pelvic ganglions, smooth muscle as well as endothelial cells of the corpora cavernosa [6, 22]. In this study, elevated estradiol levels were also observed in some patients affected by hypogonadism. It has been hypothesized that high level of estradiol might reduce NO-mediated cavernosal smooth muscle relaxation and intracavernosal pressure, worsening the erectile function [23].

Interestingly, we observed that the most frequent alteration in the group of subjects with known pathology was DM, differently from the group of subjects with new diagnosis. This could be explained by the fact that more frequently an occasional finding of hyperglycemia can occur during routine examinations. In addition, diabetes can manifest itself through different signs and symptoms before the diagnosis of ED. On the other hand, the SUBITO-DE study highlighted a high prevalence of ED, hypogonadism and depressive symptoms among male patients with newly diagnosed T2DM [24]. On this matter, psychological factors are strictly related to ED and are seldom investigated also in DM patients [25]. Furthermore, some comorbidities closely associated with diabetes as well as DM complication, such as obesity, hypertension and its pharmacological treatments, atherosclerosis, neuropathy, nephropathy, as well as infections, disease of penile structure and depression could be considered as co-factors for ED [10, 26]. This evidence is confirmed by the fact that metabolic disorders are associated with reduced T levels, arteriogenic ED and higher risk of major adverse cardiovascular events [6, 23, 27]. To this regard, Corona et al. [14] showed a higher risk of severe ED and pathological penile Doppler ultrasound parameters in patients with DM, both established and newly diagnosed, as well as impaired fasting glucose.

However, also the prevalence of newly diagnosed glycemic disorders in the present population is fairly high (17.3%). In this study, men with DM represents the group with the most severe form of ED, considering the difficulty in the achievement of erection and the absence of spontaneous erection. In fact, the severity of ED increases with the duration of diabetes, poor glycemic control and presence of microvascular complications [11], and diabetes treatment as well as educational therapy and lifestyle change should be suggested [28, 29]. However, the logistic regression analysis showed that, along with the new diagnosis of DM, hypertension and age worsen the severity of ED, but not BMI. In this regard, a recent study conducted by Yuan et al. highlighted that DM showed a direct causal effect on ED, independent of obesity and dyslipidemia [30].

A number of epidemiological data support the relationship between sexual function and testosterone levels as well as glycemic disorders [31, 32]. To this regard, Maseroli et al. showed that DM, hypogonadism and hyperprolactinemia are more frequent in subject consulting for ED compared to general population of the same geographic area [31]. However, several other hormones are also involved in sexual functioning and should be investigated in a proper work-out of ED [32]. Considering thyroid function, in this study an alteration was assess in 12.3% of cases. These data are partially in agreement with Gabrielson et al. who reported a prevalence estimated in selected populations of sexual dysfunction of about 60% in patients with hypothyroidism and hyperthyroidism [33]. In the present study, a subclinical hyperthyroidism was mainly diagnosed. Similarly, Chen et al. [34] observed an increased prevalence of ED in patients with subclinical hypothyroidism compared to patients with euthyroidism, and they recommended screening for thyroid dysfunction in men with ED. Even if hyperthyroidism was mainly associated with acquired premature ejaculation [35], in this study most of the thyroid dysfunction was related to hyperthyroidism or subclinical hyperthyroidism (10% and 55%, respectively), compared to overt hypothyroidism or subclinical hypothyroidism (10% and 24%, respectively). This suggests the need to better investigate this topic.

The prevalence of hyperprolactinemia in the present study was 11.5%. In men consulting for sexual dysfunction, hypoprolactinemia and hyperprolactinemia have been evaluated [36, 37]. However, while the association between hyperprolactinemia and hypoactive sexual desire is well defined, more studies are needed to completely understand the role of these hormones in regulating male sexual functioning. Furthermore, erectile dysfunction in association with high prolactin levels is frequent in patients suffering from psychiatric disorders and taking psychotropic drugs [38, 39]. To this regard, in this study psychiatric patients were excluded.

Finally, almost half of the patients with new diagnosis of endocrine or glycemic disorders previously used PDE5-i. This can cause a significant number of delay or missed diagnoses of the disease which caused this symptom [15].

The main limitation in this study is its retrospective nature and the absence of psychological assessment and psychometric test. Furthermore, we only included hormonal and glycemic alteration diagnosed by routine screening, therefore adrenal, parathyroid and other pituitary dysfunctions were not considered. However, no definite evidence of these last hormones, as well as for dihydrotestosterone, dehydroepiandrosterone, dehydroepiandrosterone sulfate, are available in the management of ED [32].

In conclusion, to screen endocrine disorders is very useful in the diagnostic-therapeutic management of ED patients, because it allows to define the diagnosis and establish an etiopathogenetic treatment in a large number of patients. As most endocrine causes of ED are treatable, every effort should be made to exclude potential hormonal and metabolic etiologies underlying ED at an early stage. Finally, it is still more relevant to early detect DM as it is associated with ED severity, especially in elderly man and in presence of hypertension.
